# Global hyperperfusion after successful endovascular thrombectomy is linked to worse outcome in acute ischemic stroke

**DOI:** 10.1038/s41598-024-60623-4

**Published:** 2024-05-01

**Authors:** Wookjin Yang, Jeong-Min Kim, Chul-Ho Sohn, Matthew Chung, Youngjoon Kim, Jiyeon Ha, Dong-Wan Kang, Eung-Joon Lee, Han-Yeong Jeong, Keun-Hwa Jung, Seung-Hoon Lee

**Affiliations:** 1 Department of Neurology, Seoul National University Hospital, Seoul National University College of Medicine, 101 Daehak-ro, Jongno-gu, Seoul, 03080 Korea; 2 Department of Radiology, Seoul National University Hospital, Seoul National University College of Medicine, Seoul, Republic of Korea; 3 Department of Neurology, Seoul National University Bundang Hospital, Seongnam, Republic of Korea

**Keywords:** Neurology, Cerebrovascular disorders, Stroke

## Abstract

Patients with stroke may develop hyperperfusion after a successful endovascular thrombectomy (EVT). However, the relationship between post-EVT hyperperfusion and clinical outcomes remains unclear and requires further clarification. We reviewed consecutive patients with anterior circulation occlusion who were successfully recanalized with EVT. Based on post-EVT arterial spin-labeling images, hyperperfusion was categorized as follows: global hyperperfusion (GHP), increased cerebral blood flow (CBF) in ≥ 50% of the culprit vessel territory; focal hyperperfusion (FHP), increased CBF in < 50% of the culprit vessel territory; no hyperperfusion (NHP), no discernible CBF increase. Factors associated with hyperperfusion were assessed, and clinical outcomes were compared among patients under different hyperperfusion categories. Among 131 patients, 25 and 40 patients developed GHP and FHP, respectively. Compared to other groups, the GHP group had worse National Institutes of Health Stroke Scale score (GHP vs. NHP/FHP, 18.1 ± 7.4 vs. 12.3 ± 6.0; p < 0.001), a larger post-EVT infarct volume (98.9 [42.3–132.7] vs. 13.5 [5.0–34.1] mL; p < 0.001), and a worse 90-day outcome (modified Rankin Scale, 3 [1–4] vs. 2 [0–3]; p = 0.030). GHP was independently associated with infarct volume (B = 0.532, standard error = 0.163, p = 0.001), and infarct volume was a major mediator of the association of GHP with unfavorable outcomes (total effect: β = 0.176, p = 0.034; direct effect: β = 0.045, p = 0.64; indirect effect: β = 0.132, p = 0.017). Patients presenting with post-EVT GHP had poorer neurological prognosis, which is likely mediated by a large infarct volume.

## Introduction

Cerebral hyperperfusion syndrome is a well-known complication after carotid revascularization or bypass surgery; cerebral hyperperfusion syndrome may lead to serious clinical conditions, such as brain edema and intracranial hemorrhage, or death^1,2^. Physicians and surgeons are usually well aware of this complication and take active preventive measures, such as strict postoperative blood pressure control^3–5^. However, hyperperfusion after endovascular thrombectomy (EVT) has not been thoroughly investigated, and previous studies report conflicting results. Some reported a higher chance of hemorrhagic transformation and unfavorable neurological outcomes in patients with post-EVT hyperperfusion^6–9^, whereas other studies correlated post-EVT hyperperfusion with favorable 90-day outcomes^10^.

The clinical impact of hyperperfusion after EVT may vary according to its extent; however, perfusion imaging using contrast agents after a successful EVT could be an overelaborating effort during an unstable period. Perfusion mapping with arterial spin-labeling (ASL) is a noninvasive imaging modality that can quantitatively measure tissue perfusion without using contrast material^11^. Using an ASL perfusion map embedded in routine brain MR images, we investigated the association between post-EVT hyperperfusion extent and neurological outcomes.

## Methods

### Study population

The Seoul National University Hospital (SNUH) is one of the tertiary referral centers in Korea; the SNUH Stroke Registry is an ongoing prospective stroke registry that enrolls patients with acute stroke admitted within 7 days to SNUH. Using data from this registry, we reviewed consecutive cases of patients with acute ischemic stroke who underwent EVT for anterior circulation occlusion (i.e., internal carotid artery and M1 or M2 segment of the middle cerebral artery occlusions) between January 2015 and June 2022. Among them, we selected patients who achieved successful endovascular recanalization (modified treatment in cerebral infarction grade 2b or 3)^12^. Patients with pre-stroke modified Rankin Scale (mRS) 3–5, those without available ASL perfusion images after EVT, and those lost to follow-up were excluded. This study was performed in line with the principles of the Declaration of Helsinki. The Institutional Review Board of the Seoul National University Hospital (No. 1009-062-332) approved and waived informed consent for this study, due to the retrospective nature.

### Clinical information

Data on sex, age, hypertension, diabetes, hyperlipidemia, smoking history, coexisting active cancer, initial blood pressure, initial National Institutes of Health Stroke Scale (NIHSS) score, stroke etiology, and intravenous thrombolysis use were collected at the time of index stroke for all patients included in the study. The site of culprit vessel occlusion and laterality were assessed using preprocedural CT, MR, or conventional angiography. Onset-to-reperfusion, puncture-to-reperfusion, and reperfusion-to-ASL time intervals were obtained. We obtained the following outcome data: hemorrhagic transformation, infarct volume, discharge NIHSS score, and 90-day mRS. Patients were classified as having hemorrhagic transformation if they had hemorrhagic infarction type 1 or a more severe form, per European Cooperative Acute Stroke Study II grades^13^. Unfavorable 90-day outcomes were defined as 90-day mRS scores of 3–6.

### Imaging protocols and analysis

During admission, all patients were followed up with at least one MRI after EVT. MR images were obtained on a 1.5 T (Signa HDxt, GE, Milwaukee, WI, USA [n = 10]; Ingenia, Philips, Best, The Netherlands [n = 7]) or a 3.0 T MR scanner (Discovery MR750W, GE [n = 68]; IngeniaCX, Philips [n = 21]; Verio, Siemens, Erlangen, Germany [n = 13]; Magnetom Skyra, Siemens [n = 12]) using an eight-channel or 32-channel head coil. The MRI protocol included diffusion-weighted imaging (DWI), fluid-attenuated inversion recovery (FLAIR), susceptibility-weighted imaging (SWI), three-dimensional time-of-flight (3D TOF) MR angiography, and ASL perfusion images. Table S1 summarizes the specific MRI parameters of these sequences.

**Figure 1:**
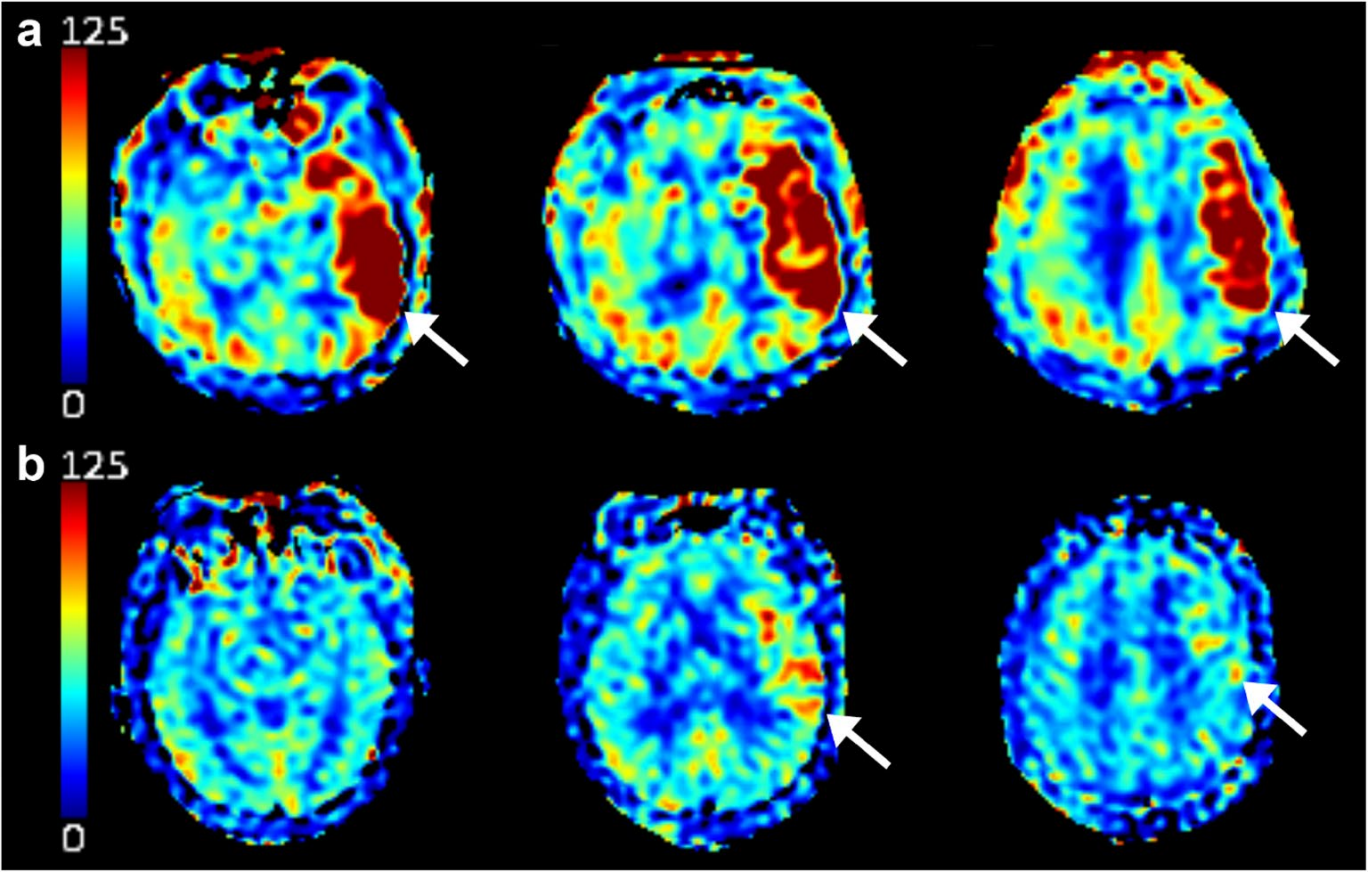
Representative ASL perfusion images of patients with GHP and FHP after a successful EVT. (**a**) Post-EVT ASL perfusion images obtained after successful recanalization of the left distal internal carotid artery occlusion in a 57-year-old man with GHP. (**b**) Post-EVT ASL perfusion images obtained after successful recanalization of the left middle cerebral artery M1 segment occlusion in a 72-year-old man with FHP. White arrows indicate areas of hyperperfusion. The color bar on the left side indicates the estimated cerebral blood flow. *ASL* arterial spin-labeling, *EVT* endovascular treatment, *FHP* focal hyperperfusion, *GHP* global hyperperfusion.

Hyperperfusion was defined as a visually discernible increase in cerebral blood flow (CBF) in the culprit vessel territory compared to the contralateral counterparts; the presence of hyperperfusion was assessed using the first ASL perfusion maps captured after reperfusion. Based on the degree of hyperperfusion, we classified patients into three categories as follows: global hyperperfusion (GHP), increased ipsilateral CBF on ≥ 50% of the culprit vessel territory; focal hyperperfusion (FHP), increased ipsilateral CBF on < 50% of the culprit vessel territory; and no hyperperfusion (NHP), no recognizable increase in ipsilateral CBF. Representative examples of GHP and FHP are shown in Fig. 1. The presence of hemorrhagic transformation was qualitatively assessed based on the last SWI images obtained upon admission. Infarct volume was calculated with a semi-automated method using the 3D Slicer software (v5.0.2, http://slicer.org) based on the last FLAIR (n = 125) or DWI (n = 6) images captured during admission. Briefly, infarcted area was manually segmented on FLAIR or DWI images with the assistance of the level tracing tool in 3D Slicer. The software then automatically determined the volume of the outlined lesion. The presence of hyperperfusion and hemorrhagic transformation was evaluated by two experienced neurologists (WY, 10 years of experience and J-MK, 20 years of experience) blinded to the clinical and imaging data except for information on the culprit vessel. They were also blinded to each other’s rating. A senior neuroradiologist (C-HS, 35 years of experience) was consulted to achieve consensus in cases where there was a disagreement.

For sensitivity analysis, we investigated the relationship between pre-EVT tissue ischemia from CT perfusion and hyperperfusion status from post-EVT brain MRI. Pre-EVT infarct core and penumbra volumes were available in approximately two-thirds of the study population, as shown by CT perfusion maps. CT images were obtained on a multidetector row scanner (Aquilion ONE, Toshiba, Tokyo, Japan) using the following scan settings: tube voltage, 80 kVp; tube current, 150 mA; slice thickness, 5 mm. A non-ionic contrast agent (50 mL, Iomeron 400 mL, Brocca, Milan, Italy) was injected at 5 mL/s using a power injector, followed by a 30 mL saline flush. The infarct core and penumbra volumes were estimated using Vitrea software (Vital Images, MN, USA) and the singular value decomposition plus algorithm. Voxels that exceeded both the relative cerebral blood volume (rCBV) and time-to-peak (TTP) thresholds were considered as the infarct core. Voxels that exceeded the TTP threshold were considered to be the penumbra. The threshold for rCBV was set at 41% reduction with a 3% interval. The threshold for TTP was set at 6.8 s with a 0.5-s interval^14^.

### Statistical analysis

Continuous variables were compared using one-way ANOVA, Kruskal–Wallis test, *t*-test, or Mann–Whitney U test, as appropriate. Categorical variables were compared using the chi-square test or Fisher’s exact test, as appropriate. The interrater agreements for the degree of hyperperfusion and the presence of hemorrhagic transformation were assessed using the kappa statistics. Pairwise comparisons of the baseline parameters among the three groups, based on the degree of hyperperfusion, were performed using the Bonferroni method. Bivariate analyses, utilizing Pearson’s correlation test, Kruskal–Wallis test, or Mann–Whitney *U* test, assessed potential associations between baseline clinicoradiological factors and outcomes, including infarct volume and 90-day mRS score. Age, sex, and variables with p < 0.15 in the bivariate analyses were included in the following multivariate and mediation analyses as potential confounders. To assess the association between hyperperfusion groups and outcome data, linear regression for infarct volume and binary logistic regression for unfavorable 90-day outcomes were conducted. Variance inflation factors were calculated to assess multicollinearity between variables in each model. Mediation analysis was performed to assess the potential mediating effect of infarct volume on the relationship between GHP and 90-day mRS. The bootstrapping method with 1000 resamples was used to estimate 95% confidence intervals. To elucidate the mechanisms underlying post-EVT GHP, pre-EVT core and penumbra volumes were compared across hyperperfusion groups using the Kruskal–Wallis test. Two-sided probability values of < 0.05 were considered statistically significant. Statistical analyses were performed using R (v4.1.3, R Foundation, Vienna, Austria).

## Results

**Figure 2:**
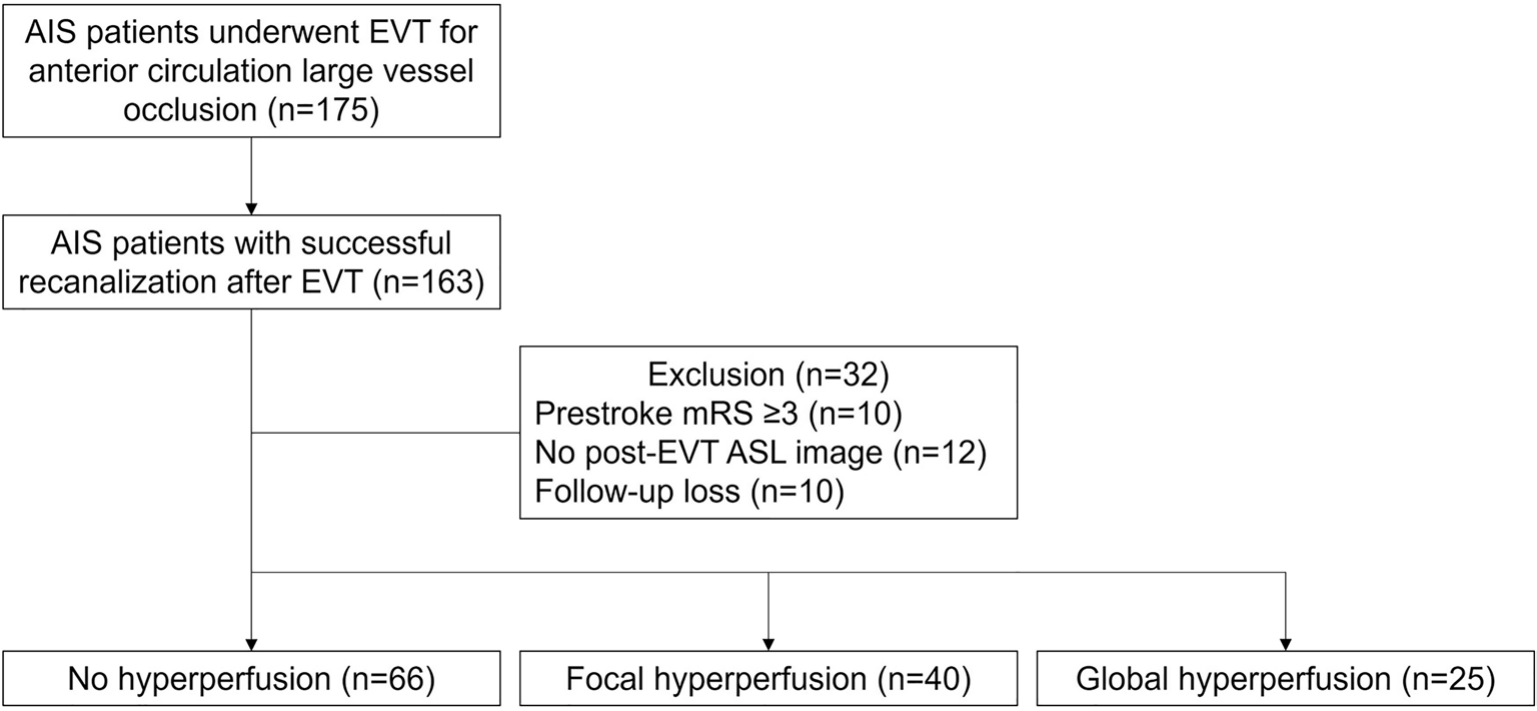
Flowchart of the patient inclusion and exclusion. *AIS* acute ischemic stroke, *ASL* arterial spin-labeling, *EVT* endovascular treatment, *mRS* modified Rankin Scale.

A total of 131 patients were included based on the pre-specified criteria (Fig. 2). The median [interquartile range] age was 70 [61–77.5] years, and 71 (54.2%) were men. The median [interquartile range] time intervals from reperfusion to ASL imaging and to infarct volume measurements were 1 [1–3] and 2 [1–4] days, respectively. Twenty-eight patients were available for additional MR images obtained after post-EVT ASL imaging; in these patients, infarct volume was measured based on follow-up FLAIR or DWI images (median [interquartile range] time interval from ASL to infarct volume measurement, 3.5 [2–5] days). The infarct volume of the remaining 103 patients was assessed based on MR images captured simultaneously with ASL images. Among the 131 patients, 25 (19.1%) and 40 (30.5%) patients developed post-EVT GHP and FHP, respectively. The remaining 66 patients (50.4%) were categorized as NHP. The interrater agreements were good for the degree of hyperperfusion (weighted Cohen’s kappa, 0.75; 95% CI, 0.66–0.83), and excellent for the presence of hemorrhagic transformation (Cohen’s kappa, 0.82; 95% CI, 0.72–0.92).

**Figure 3:**
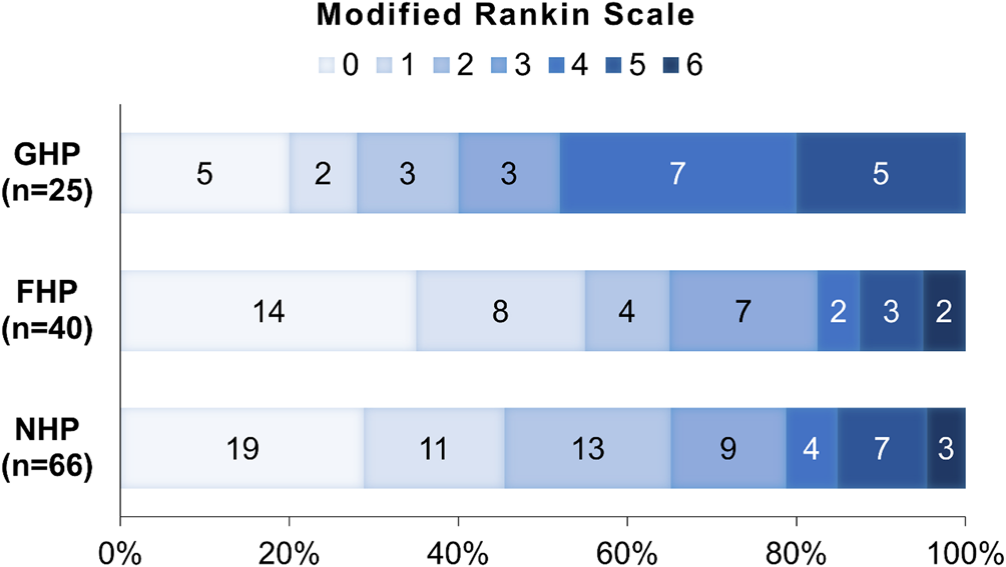
Distribution of modified Rankin Scale at 90 days according to the degree of post-EVT hyperperfusion. *EVT* endovascular treatment, *FHP* focal hyperperfusion, *GHP* global hyperperfusion, *NHP* no hyperperfusion.

Patients with GHP presented with more severe neurological deficits (initial NIHSS score, GHP vs. NHP/FHP, mean ± standard deviation, 18.1 ± 7.4 vs. 12.3 ± 6.0; p < 0.001) than those with NHP or FHP. The initial diastolic blood pressure was lower in the GHP group than that in the NHP group. However, no demonstrable differences in demographic factors, risk factor profiles, or index stroke characteristics were found between the FHP and NHP groups. Patients with GHP had larger infarct volumes (GHP vs. NHP/FHP, median [interquartile range], 98.9 [42.3–132.7] vs. 13.5 [5.0–34.1] mL; p < 0.001) and worse neurological outcomes, including discharge NIHSS score (9 [3–14] vs. 3 [0–6]; p = 0.001) and 90-day mRS (3 [1–4] vs. 2 [0–3]; p = 0.030), than those with NHP or FHP. No significant difference in hemorrhagic transformation was found according to the degree of hyperperfusion (Table 1, Tables S2, and S3; Fig. 3).

**Table 1:** Baseline characteristics and clinical outcomes according to the presence of post-EVT GHP.

	NHP/FHP (n = 106)	GHP (n = 25)	p value
Age	70 [59–77]	70 [62–78]	0.71
Male sex	55 (51.9%)	16 (64.0%)	0.38
Body mass index	23.7 ± 3.1	22.9 ± 3.0	0.21
Hypertension	72 (67.9%)	17 (68.0%)	> 0.99
Diabetes	38 (35.8%)	4 (16.0%)	0.094
Hyperlipidemia	54 (50.9%)	7 (28.0%)	0.065
Ever smoking	22 (20.8%)	6 (24.0%)	0.93
Active cancer	14 (13.2%)	3 (12.0%)	> 0.99
Prestroke mRS			0.89
0	89 (84.0%)	21 (84.0%)	
1	14 (13.2%)	3 (12.0%)	
2	3 (2.8%)	1 (4.0%)	
Initial systolic blood pressure	147.5 [132–177]	140 [135–152]	0.11
Initial diastolic blood pressure	81 [70–93]	73 [70–83]	0.042
Initial NIHSS	12.3 ± 6.0	18.1 ± 7.4	< 0.001
Occlusion site			0.079
ICA or M1	84 (79.2%)	15 (60.0%)	
M2	22 (20.8%)	10 (40.0%)	
Left side occlusion	54 (50.9%)	15 (60.0%)	0.55
Stroke etiology			0.80
LAA	20 (18.9%)	4 (16.0%)	
CE	60 (56.6%)	16 (64.0%)	
Others	26 (24.5%)	5 (20.0%)	
Intravenous thrombolysis	33 (31.1%)	13 (52.0%)	0.083
Recanalization state			0.53
mTICI 2b	41 (38.7%)	12 (48.0%)	
mTICI 3	65 (61.3%)	13 (52.0%)	
Onset-to-reperfusion time, minutes	413.5 [201–593]	320 [197–489]	0.32
Puncture to reperfusion, minutes	40 [24–60]	42 [22–53]	0.81
Reperfusion to ASL interval, days	1 [1–3]	1 [1–4]	0.75
Hemorrhagic transformation	50 (47.2%)	14 (56.0%)	0.57
Infarct volume, mL	13.5 [5.0–34.1]	98.9 [42.3–132.7]	< 0.001
Discharge NIHSS	3 [0–6]	9 [3–14]	0.001
90-day mRS	2 [0–3]	3 [1–4]	0.030
Unfavorable 90-day outcome	37 (34.9%)	15 (60.0%)	0.038

Data are presented as means ± standard deviations, medians [interquartile ranges], or number (%).

*ASL* arterial spin-labeling, *CE* cardioembolism, *EVT* endovascular treatment, *FHP* focal hyperperfusion, *GHP* global hyperperfusion, *ICA* internal carotid artery, *LAA* large artery atherosclerosis, *mRS* modified Rankin Scale, *mTICI* the modified treatment in cerebral infarction, *NHP* no hyperperfusion, *NIHSS* National Institutes of Health Stroke Scale.

**Figure 4:**
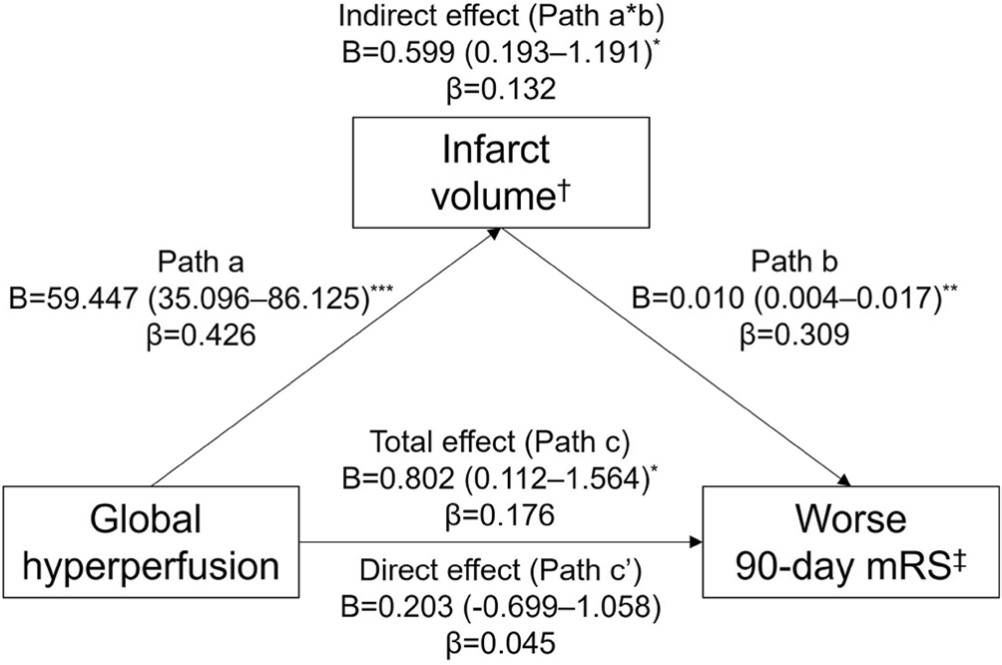
Mediating role of infarct volume on the effect of global hyperperfusion on worse 90-day mRS. Unstandardized and standardized regression coefficients for each path of the mediation model are presented. The 95% bootstrap confidence intervals for the unstandardized coefficients are provided. ^†^Controlled for age, sex, smoking, initial NIHSS score, occlusion site of M2, intravenous thrombolysis, and hemorrhagic transformation. ^‡^Controlled for age, sex, hypertension, active cancer, initial diastolic blood pressure, initial NIHSS score, stroke etiology, intravenous thrombolysis, and hemorrhagic transformation. ***p < 0.001; **p < 0.01; *p < 0.05. *B* unstandardized coefficient, *β* standardized coefficient, *mRS* modified Rankin Scale, *NIHSS* National Institutes of Health Stroke Scale.

GHP was associated with infarct volume, whereas FHP was not. GHP showed an independent association with infarct volume in the subsequent multivariate analysis, controlling for potential confounders (Table 2; Tables S4, and S5 for bivariate analyses). Univariate analysis showed a significant association between GHP and unfavorable 90-day outcomes; however, this association was attenuated after adjusting for infarct volume (Table 3; Tables S4, and S5 for bivariate analyses). In the mediation analysis, the indirect effect of post-EVT GHP on worse 90-day mRS scores via infarct volume was significant; however, no demonstrable direct effect was observed. Thus, infarct volume completely mediated the relationship between GHP and worse 90-day mRS scores (Fig. 4).

**Table 2:** Linear regression analysis of factors associated with infarct volume.

	Univariate analysis			Multivariate analysis^†^		
	B (SE)	β	p value	B (SE)	β	p value
Age	0.004 (0.005)	0.061	0.49	− 0.001 (0.004)	− 0.024	0.73
Male sex	0.146 (0.143)	0.090	0.31	0.045 (0.126)	0.028	0.72
Ever smoking	0.406 (0.170)	0.205	0.019	0.261 (0.154)	0.132	0.092
Initial NIHSS	0.052 (0.010)	0.423	< 0.001	0.031 (0.009)	0.253	0.001
M2 occlusion^‡^	− 0.387 (0.162)	− 0.206	0.019	− 0.400 (0.137)	− 0.212	0.004
Intravenous thrombolysis	0.429 (0.145)	0.253	0.004	0.306 (0.120)	0.181	0.012
Hemorrhagic transformation	0.618 (0.132)	0.382	< 0.001	0.515 (0.115)	0.318	< 0.001
Hyperperfusion						
No hyperperfusion	Reference			Reference		
Focal hyperperfusion	0.181 (0.153)	0.103	0.24	0.069 (0.129)	0.039	0.59
Global hyperperfusion	0.799 (0.179)	0.388	< 0.001	0.532 (0.163)	0.258	0.001

^†^R^2^ = 0.395 and p < 0.001 for the linear regression equation. The multivariate model incorporated the following variables: age, sex, smoking, initial NIHSS score, occlusion site, intravenous thrombolysis, hemorrhagic transformation, and the degree of hyperperfusion.

^‡^Internal carotid artery and M1 occlusions were grouped together versus M2 occlusion.

*B* unstandardized coefficient, *β* standardized coefficient, *NIHSS* National Institutes of Health Stroke Scale, *SE* standard error.

**Table 3:** Binary logistic regression analysis for the relationship between hyperperfusion and unfavorable 90-day outcomes.

	Univariate analysis		Multivariate analysis^†^	
	OR (95% CI)	p value	OR (95% CI)	p value
Age	1.03 (1.00–1.06)	0.029	1.05 (1.00–1.09)	0.036
Male sex	0.66 (0.33–1.34)	0.26	0.64 (0.26–1.59)	0.34
Hypertension	2.41 (1.08–5.38)	0.032	1.74 (0.60–5.03)	0.31
Active cancer	1.86 (0.67–5.18)	0.24	2.64 (0.64–10.91)	0.18
Initial diastolic blood pressure	1.02 (1.00–1.04)	0.095	1.02 (0.99–1.06)	0.14
Initial NIHSS	1.11 (1.05–1.18)	< 0.001	1.05 (0.97–1.14)	0.22
Stroke etiology				
LAA	Reference		Reference	
CE	2.62 (0.88–7.76)	0.082	3.09 (0.85–11.19)	0.086
Others	4.05 (1.21–13.61)	0.024	5.07 (1.13–22.69)	0.034
Intravenous thrombolysis	2.56 (1.22–5.35)	0.013	1.73 (0.70–4.30)	0.24
Hemorrhagic transformation	2.69 (1.31–5.54)	0.007	1.51 (0.58–3.95)	0.40
Infarct volume	3.64 (1.95–6.80)	< 0.001	2.66 (1.08–6.54)	0.034
Hyperperfusion				
No hyperperfusion	Reference		Reference	
Focal hyperperfusion	1.01 (0.44–2.29)	0.99	1.10 (0.40–3.05)	0.85
Global hyperperfusion	2.80 (1.09–7.23)	0.033	1.54 (0.38–6.29)	0.55

^†^The multivariate model incorporated the following variables: age, sex, hypertension, active cancer, initial diastolic blood pressure, initial NIHSS score, stroke etiology, intravenous thrombolysis, hemorrhagic transformation, infarct volume, and the degree of hyperperfusion.

*CI* confidence interval, *NIHSS* National Institutes of Health Stroke Scale, *OR* odds ratio.

Data on pre-EVT estimated core and penumbra volumes based on the Vitrea software were available for 89 (67.9%) of 131 patients. Out of the 89 patients, 15, 23, and 51 patients developed GHP, FHP, and NHP. The time interval between the last known well time and CT scan was similar between the three groups (GHP vs. FHP vs. NHP, median [interquartile range], 198.5 [125–358] vs. 147.5 [76–419] vs. 205 [84.5–554.5] minutes; p = 0.61). Estimated core (98.9 [42.3–132.7] vs. 11.1 [6.7–32.8] vs. 15.2 [3.1–34.1]; p < 0.001) and penumbra volumes (122.0 [58.3–177.0] vs. 56.8 [41.3–103.8] vs. 66.3 [30.1–97.6]; p = 0.035) were significantly higher in the GHP group than those in the NHP or FHP groups.

## Discussion

Post-EVT hyperperfusion was frequently observed in patients with stroke who underwent successful recanalization. Patients with GHP, but not those with FHP, had larger post-EVT infarct volumes and worse clinical outcomes than patients with NHP. Poor prognosis in the GHP group appeared to be primarily driven by a larger infarct volume rather than an independent effect of GHP. Patients with GHP had a significantly larger pre-EVT core volume.

More severe neurological symptoms, higher pre-EVT core volume, and larger post-EVT infarct volume in the GHP group may indicate that post-EVT GHP is an indicator of a poor collateral status. In patients with stroke who underwent EVT, hyperperfusion may be mainly observed around the area of blood–brain barrier disruption, which is likely to be broader in patients with larger ischemic core^15^. Following this, luxury perfusion occurring in this broader area with a disrupted blood–brain barrier may present as GHP in brain imaging. Namely, compromised collateral circulation in patients with GHP may be responsible for their larger pre-EVT core volume formed at a similar time interval^16–18^, compared to that of the other two groups. Considering the overall results of the mediation analysis, post-EVT GHP may be a prognostic marker of unfavorable outcomes reflecting poor collateral status.

The contradictory findings of recent studies regarding the effect of post-EVT hyperperfusion on clinical outcomes appear to be the consequence of different inclusion criteria. One study suggested that hyperperfusion was associated with good prognosis included patients with stroke who underwent EVT, regardless of post-EVT recanalization status. In the study, the presence of hyperperfusion reflected successful recanalization and was subsequently associated with favorable outcomes^10^. In contrast, in other studies that included only successfully recanalized patients, post-EVT hyperperfusion was associated with a higher chance of hemorrhagic transformation and worse neurological outcomes^6,8,9^. Our findings agree with those of the latter study, supporting that post-EVT hyperperfusion is an indicator of poor outcome among patients with stroke who underwent successful recanalization. Given the great impact of recanalization status on clinical outcomes^19^, it would be more appropriate to exclude patients with failed EVT to assess the effect of hyperperfusion, similar to the latter study and our study.

The effectiveness of the management of post-EVT GHP in improving prognosis should be further addressed. Our findings suggest that poor prognosis in patients with post-EVT GHP may be attributable mainly to the larger pre-EVT core and resulting larger infarct volume. From this perspective, strict blood pressure management might not be as effective for patients with post-EVT GHP after acute ischemic stroke as it is for patients with cerebral hyperperfusion syndrome following revascularization for chronic ischemia. Maintaining blood pressure within an adequate range may be more helpful rather than strictly lowering it in patients with post-EVT GHP, considering potential relationship between GHP and poor collateral status^20^. A recent report of the harm of more intensive blood pressure lowering after successful EVT provides support for this hypothesis^21^. Optimal blood pressure control strategy after recanalization requires future studies, and ASL mapping can be utilized to select at-risk populations in such clinical studies to enhance post-EVT prognosis.

This study had several limitations. First, although we analyzed a larger number of patients than other studies on this topic, the sample size was still insufficient to allow robust generalization of the results, particularly for patients in the GHP group. Second, while we suggested a potential link between poor collateral status and the association of GHP and unfavorable outcomes, it was not possible to incorporate reliable markers of collateral status, such as the hypoperfusion intensity ratio, in our analysis due to limited accessibility. Third, it is possible that the ASL sequence may not be widely available in stroke centers. Finally, we could not investigate the difference in the relationship between GHP and clinical outcomes according to stroke etiology. A previous study suggested that post-EVT hyperperfusion might have different underlying pathomechanisms by stroke subtype^7^; however, nearly 60% of the participants had cardioembolic stroke and the number of patients with large artery atherosclerosis stroke who developed post-EVT GHP was small (n = 4) in our study. Future studies that separately analyze the impact of post-EVT hyperperfusion by stroke etiology may provide deeper insights into its pathomechanism.

Post-EVT hyperperfusion was prevalent after successful recanalization of acute ischemic stroke. GHP that develops after successful EVT may be indicative of larger post-EVT infarct volumes, which could subsequently result in worse functional outcomes. The optimal management strategy for patients who develop post-EVT GHP requires further elucidation.

### Supplementary Information

Supplementary Tables.

## Data Availability

The data supporting the findings of this study are available from the corresponding author upon reasonable request.
