# Supplementary material for: Global hyperperfusion after successful endovascular thrombectomy is linked to worse outcome in acute ischemic stroke
Source: Sci Rep. 2024 May 1;14:10024. doi: 10.1038/s41598-024-60623-4 (PMC11063193; doi:10.1038/s41598-024-60623-4)
Supplement: Supplementary file 1 — Supplementary Tables. [file 41598_2024_60623_MOESM1_ESM.docx]

**Table S1. Magnetic resonance imaging parameters**

| **Parameters** | **DWI** | **FLAIR** | **SWI** | **Pseudocontinuous ASL-PWI**^†^ | **Pulsed ASL-PWI**^‡^ | **TOF MRA** |
| --- | --- | --- | --- | --- | --- | --- |
| Slice numbers | 37–40 | 24–30 | 46–112 | 20–64 | 12 | 133–172 |
| Slice thickness (mm) | 3–3.6 | 4­–5 | 2–3 | 5–6 | 5 | 0.6–1.2 |
| Interslice gap (mm) | 0.4–1 | 0.8–1.2 | 0 | 0 | 0 | 0 |
| Field of view (mm) | 240 × 240 | 199–220 × 220 | 199–240 × 220–240 | 240 × 240 | 240 × 240 | 179–220 × 210–240 |
| Matrix | 128­–180 × 122­–177 | 288–384 × 181–288 | 304–480 × 256–318 | 128 × 128 or 64 × 60 | 64 × 46 | 320–580 × 192–323 |
| Repetition time (msec) | 5245.6–9800 | 8000–9000 | 29–52 | 3849.5–7247 | 4600 | 18–27 |
| Echo time (msec) | 65­–87.2 | 91.5–124.2 | 0–21.3 | 9.4–11 | 17.2 | 3.4–6.9 |
| Flip angle (degree) | 90 or 180 | 90–150 | 15–30 | 90–155 | 180 | 18–25 |

^†^ Pseudocontinuous ASL-PWI was obtained using a 1.5T (Signa HDxt, GE [n=10]; Ingenia, Philips [n=7]) or a 3.0T (Discovery MR750W, GE [n=68]; IngeniaCX, Philips [n=21]) MR scanner. Data were acquired with a post-labeling delay of 1525 millisecond and label duration of 1500 millisecond.

^‡^ Pulsed ASL-PWI was obtained on a 3.0T (Verio, Siemens [n=13]; Magnetom Skyra, Siemens [n=12]) MR scanner. The bolus and inversion times were 700 and 1100 milliseconds, respectively.

ASL-PWI, arterial spin-labeling perfusion-weighted imaging; DWI, diffusion-weighted imaging; FLAIR, fluid-attenuated inversion recovery; SWI, susceptibility-weighted imaging; TOF MRA, time-of-flight magnetic resonance angiography

**Table S2. Baseline characteristics and clinical outcomes according to the degree of post-EVT hyperperfusion**

|  | **NHP (n=66)** | **FHP (n=40)** | **GHP (n=25)** | **p value** |
| --- | --- | --- | --- | --- |
| Age | 71 [56; 78] | 68 [62; 74] | 70 [62; 78] | 0.87 |
| Male sex | 35 (53.0%) | 20 (50.0%) | 16 (64.0%) | 0.53 |
| Body mass index | 23.8 ± 2.8 | 23.7 ± 3.7 | 22.9 ± 3.0 | 0.45 |
| Hypertension | 46 (69.7%) | 26 (65.0%) | 17 (68.0%) | 0.88 |
| Diabetes | 23 (34.8%) | 15 (37.5%) | 4 (16.0%) | 0.15 |
| Hyperlipidemia | 33 (50.0%) | 21 (52.5%) | 7 (28.0%) | 0.11 |
| Ever smoking | 14 (21.2%) | 8 (20.0%) | 6 (24.0%) | 0.93 |
| Active cancer | 7 (10.6%) | 7 (17.5%) | 3 (12.0%) | 0.61 |
| Prestroke mRS |  |  |  | 0.78 |
| 0 | 54 (81.8%) | 35 (87.5%) | 21 (84.0%) |  |
| 1 | 9 (13.6%) | 5 (12.5%) | 3 (12.0%) |  |
| 2 | 3 (4.5%) | 0 (0.0%) | 1 (4.0%) |  |
| Initial systolic blood pressure | 146.5 [133; 175] | 148 [132; 180] | 140 [135; 152] | 0.26 |
| Initial diastolic blood pressure | 82 [71; 96] | 77.5 [69.5; 90] | 73 [70; 83] | 0.059 |
| Initial NIHSS | 12.2 ± 6.0 | 12.4 ± 6.1 | 18.1 ± 7.4 | <0.001 |
| Occlusion site |  |  |  | 0.040 |
| ICA or M1 | 49 (74.2%) | 35 (87.5%) | 15 (60.0%) |  |
| M2 | 17 (25.8%) | 5 (12.5%) | 10 (40.0%) |  |
| Left side occlusion | 35 (53.0%) | 19 (47.5%) | 15 (60.0%) | 0.62 |
| Stroke etiology |  |  |  | 0.69 |
| LAA | 12 (18.2%) | 8 (20.0%) | 4 (16.0%) |  |
| CE | 35 (53.0%) | 25 (62.5%) | 16 (64.0%) |  |
| Others | 19 (28.8%) | 7 (17.5%) | 5 (20.0%) |  |
| Intravenous thrombolysis | 18 (27.3%) | 15 (37.5%) | 13 (52.0%) | 0.082 |
| Recanalization state |  |  |  | 0.57 |
| mTICI 2b | 24 (36.4%) | 17 (42.5%) | 12 (48.0%) |  |
| mTICI 3 | 42 (63.6%) | 23 (57.5%) | 13 (52.0%) |  |
| Onset-to-reperfusion time, minutes | 425.5 [218; 650] | 358.5 [180.5; 562] | 320 [197; 489] | 0.27 |
| Puncture-to-reperfusion, minutes | 38 [24; 64] | 41 [20.5; 58.5] | 42 [22; 53] | 0.90 |
| Reperfusion to ASL interval, days | 1 [1; 3] | 1 [1; 3] | 1 [1; 4] | 0.88 |
| Hemorrhagic transformation | 30 (45.5%) | 20 (50.0%) | 14 (56.0%) | 0.66 |
| Infarct volume, mL | 15.2 [3.1; 34.1] | 11.1 [6.7; 32.8] | 98.9 [42.3; 132.7] | <0.001 |
| Discharge NIHSS | 3 [0; 9] | 2 [0; 6] | 9 [3; 14] | 0.002 |
| 90-day mRS | 2 [0; 3] | 1 [0; 3] | 3 [1; 4] | 0.077 |
| Unfavorable 90-day outcome | 23 (34.8%) | 14 (35.0%) | 15 (60.0%) | 0.070 |

Data are presented as means ± standard deviations, medians [interquartile ranges], or number (%).

ASL, arterial spin-labeling; CE, cardioembolism; EVT, endovascular treatment; FHP, focal hyperperfusion; GHP, global hyperperfusion; ICA, internal carotid artery; LAA, large artery atherosclerosis; mRS, modified Rankin Scale; mTICI, the modified treatment in cerebral infarction; NHP, no hyperperfusion; NIHSS, National Institutes of Health Stroke Scale.

**Table S3. Bonferroni-adjusted p-values from pairwise comparisons of covariates by the degree of hyperperfusion**

|  | **FHP vs. NHP** | **GHP vs. NHP** | **GHP vs. FHP** |
| --- | --- | --- | --- |
| Age | >0.99 | >0.99 | >0.99 |
| Male sex | >0.99 | >0.99 | >0.99 |
| Body mass index | >0.99 | 0.61 | 0.91 |
| Hypertension | >0.99 | >0.99 | >0.99 |
| Diabetes | >0.99 | 0.40 | 0.35 |
| Hyperlipidemia | >0.99 | 0.30 | 0.28 |
| Ever smoking | >0.99 | >0.99 | >0.99 |
| Active cancer | >0.99 | >0.99 | >0.99 |
| Prestroke mRS | >0.99 | >0.99 | >0.99 |
| Initial systolic blood pressure | >0.99 | 0.17 | 0.51 |
| Initial diastolic blood pressure | 0.45 | 0.034 | 0.67 |
| Initial NIHSS | >0.99 | <0.001 | 0.002 |
| Occlusion site | 0.50 | 0.85 | 0.072 |
| Laterality | >0.99 | >0.99 | >0.99 |
| Stroke etiology | >0.99 | >0.99 | >0.99 |
| Intravenous thrombolysis | >0.99 | 0.15 | >0.99 |
| Recanalization state | >0.99 | >0.99 | >0.99 |
| Onset-to-reperfusion time, minutes | 0.35 | 0.26 | >0.99 |
| Puncture to reperfusion, minutes | >0.99 | >0.99 | >0.99 |
| Reperfusion to ASL interval, days | 0.54 | >0.99 | 0.64 |
| Hemorrhagic transformation | >0.99 | >0.99 | >0.99 |
| Infarct volume, mL | >0.99 | <0.001 | <0.001 |
| Discharge NIHSS | >0.99 | 0.016 | 0.014 |
| 90-day mRS | >0.99 | 0.15 | 0.067 |
| Unfavorable 90-day outcome | >0.99 | 0.16 | 0.26 |

ASL, arterial spin-labeling; FHP, focal hyperperfusion; GHP, global hyperperfusion; mRS, modified Rankin Scale; NHP, no hyperperfusion; NIHSS, National Institutes of Health Stroke Scale

**Table S4. Bivariate analyses of continuous variables for outcomes**

|  | **Infarct volume** | | **90-day mRS** | |
| --- | --- | --- | --- | --- |
|  | **Coefficient** | **p value** | **Coefficient** | **p value** |
| Age | 0.015 | 0.86 | 0.230 | 0.008 |
| Prestroke mRS | 0.055 | 0.53 | 0.050 | 0.57 |
| Initial systolic blood pressure | 0.067 | 0.45 | 0.072 | 0.41 |
| Initial diastolic blood pressure | 0.060 | 0.49 | 0.134 | 0.13 |
| Initial NIHSS | 0.410 | <0.001 | 0.274 | 0.002 |
| Onset to reperfusion (minutes) | -0.115 | 0.19 | -0.052 | 0.56 |
| Puncture to reperfusion (minutes) | -0.033 | 0.71 | -0.072 | 0.41 |
| Reperfusion to ASL interval (days) | 0.109 | 0.22 | 0.006 | 0.95 |
| Infarct volume | . | . | 0.389 | <0.001 |

All variables were analyzed using the Pearson’s correlation test.

ASL, arterial spin-labeling; mRS, modified Rankin Scale; NIHSS, National Institutes of Health Stroke Scale.

**Table S5. Bivariate analyses of categorical variables for outcomes**

|  | **Infarct volume** | **p value** | **90-day mRS** | **p value** |
| --- | --- | --- | --- | --- |
| Sex |  | 0.18 |  | 0.082 |
| Male | 22.2 [7.1–79.6] |  | 2 [0–3] |  |
| Female | 13.5 [5.2–40.4] |  | 2 [0.5–4.5] |  |
| Hypertension |  | 0.72 |  | 0.061 |
| Yes | 18.1 [6.6;66.9] |  | 2 [0–4] |  |
| No | 16.1 [5.5–50.0] |  | 1 [0–3] |  |
| Diabetes |  | 0.99 |  | 0.30 |
| Yes | 16.9 [5.6–46.6] |  | 2 [0–4] |  |
| No | 16.2 [6.2–66.9] |  | 2 [0–3] |  |
| Hyperlipidemia |  | 0.62 |  | 0.87 |
| Yes | 16.7 [5.6–38.9] |  | 2 [0–3] |  |
| No | 17.1 [6.2–81.4] |  | 2 [0–4] |  |
| Ever smoking |  | 0.015 |  | 0.35 |
| Yes | 28.4 [13.5–105.9] |  | 1.5 [0–3] |  |
| No | 13.8 [5.5–42.5] |  | 2 [0–4] |  |
| Active cancer |  | 0.55 |  | 0.096 |
| Yes | 28.3 [8.7–42.7] |  | 3 [1–5] |  |
| No | 16.1 [5.5–77.0] |  | 2 [0–3] |  |
| Stroke etiology^†^ |  | 0.57 |  | 0.061 |
| LAA | 15.0 [6.1–52.4] |  | 1 [0–2] |  |
| CE | 13.9 [3.6–63.9] |  | 2 [0–3] |  |
| Others | 23.6 [11.0­–79.3] |  | 3 [0.5–5] |  |
| Occlusion site |  | 0.088 |  | 0.74 |
| M1 or ICA | 22.2 [6.9–72.0] |  | 2 [0–4] |  |
| M2 | 13.2 [2.4–37.1] |  | 2 [0–3] |  |
| Laterality |  | 0.42 |  | 0.30 |
| Left occlusion | 21.3 [8.4–66.9] |  | 1.5 [0–3] |  |
| Right occlusion | 15.8 [4.1–50.0] |  | 2 [0–4] |  |
| IV thrombolysis |  | 0.004 |  | 0.14 |
| Yes | 33.2 [8.4–99.2] |  | 3 [0–4] |  |
| No | 13.2 [4.1–37.7] |  | 2 [0–3] |  |
| Recanalization state |  | 0.44 |  | 0.75 |
| mTICI 2b | 17.0 [7.9–77.8] |  | 2 [0–4] |  |
| mTICI 3 | 15.6 [4.1–46.6] |  | 2 [0–3] |  |
| Hemorrhagic transformation |  | <0.001 |  | 0.002 |
| Yes | 32.3 [13.2–105.9] |  | 3 [1–4] |  |
| No | 9.9 [2.9–25.6] |  | 1 [0–3] |  |
| Hyperperfusion^†^ |  | <0.001 |  | 0.077 |
| NHP | 15.2 [3.1–34.1] |  | 2 [0–3] |  |
| FHP | 11.1 [6.7–32.8] |  | 1 [0–3] |  |
| GHP | 98.9 [42.3–132.7] |  | 3 [1–4] |  |

Data are presented as medians [interquartile ranges].

^†^ Variables analyzed using the Kruskal-Wallis test. All other variables were analyzed using the Mann-Whitney U test.

CE, cardioembolism; FHP, focal hyperperfusion; GHP, global hyperperfusion; ICA, internal carotid artery; LAA, large artery atherosclerosis; mRS, modified Rankin Scale; mTICI, modified treatment in cerebral infarction; NHP, no hyperperfusion
